# Morphological and phylogenetic analyses reveal three new species of *Diaporthe* from Yunnan, China

**DOI:** 10.3897/mycokeys.78.60878

**Published:** 2021-02-19

**Authors:** Shengting Huang, Jiwen Xia, Xiuguo Zhang, Wenxiu Sun

**Affiliations:** 1 College of Life Sciences, Yangtze University, Jingzhou 434025, Hubei, China Yangtze University Jingzhou China; 2 Shandong Provincial Key Laboratory for Biology of Vegetable Diseases and Insect Pests, College of Plant Protection, Shandong Agricultural University, Taian, Shandong, 271018, China Shandong Agricultural University Taian China

**Keywords:** Diaporthaceae, Diaporthales, phylogeny, taxonomy, three taxa new to science

## Abstract

Species of *Diaporthe* have often been reported as plant pathogens, endophytes or saprobes, commonly isolated from a wide range of plant hosts. Sixteen strains isolated from species of ten host genera in Yunnan Province, China, represented three new species of *Diaporthe*, *D. chrysalidocarpi*, *D. machili* and *D. pometiae* as well as five known species *D. arecae*, *D. hongkongensis*, *D. middletonii*, *D. osmanthi* and *D. pandanicola*. Morphological comparisons with known species and DNA-based phylogenies based on the analysis of a multigene (ITS, TUB, TEF, CAL and HIS) dataset support the establishment of the new species. This study reveals that a high species diversity of *Diaporthe* with wide host ranges occur in tropical rainforest in Yunnan Province, China.

## Introduction

The genus *Diaporthe* (DiaporthaceaeDiaporthales) with asexual morphs previously known as *Phomopsis* spp. is based on the type species *Diaporthe eres*Nitschke (1870) from *Ulmus* sp. in Germany. Rossman et al. (2015) proposed to use the name *Diaporthe* over *Phomopsis* in the context of the one fungus – one name initiative, because it was described first, is encountered commonly in literature and includes the majority of known species. The sexual morph of *Diaporthe* is characterised by immersed ascomata and an erumpent pseudostroma with elongated perithecial necks; asci are unitunicate, clavate to cylindrical; and ascospores are fusoid, ellipsoid to cylindrical, hyaline, biseriate to uniseriate in the ascus, sometimes with appendages (Udayanga et al. 2011; Senanayake et al. 2017, 2018). The asexual morph is characterised by ostiolate pycnidia with cylindrical phialides often producing three types of hyaline, aseptate conidia called α-conidia, β-conidia and γ-conidia (Udayanga et al. 2011; Gomes et al. 2013). The α-conidia and β-conidia are produced frequently, but the γ-conidia are rarely observed (Gomes et al. 2013; Guarnaccia and Crous 2017; Guo et al. 2020).

Currently, more than 1100 epithets of *Diaporthe* are listed in Index Fungorum (http://www.indexfungorum.org/; accessed 1 Nov. 2020), but only one-fifth of these taxa have been well-studied with ex-type cultures and supplementary DNA barcodes (Guo et al. 2020; Yang et al. 2020; Zapata et al. 2020). Species of *Diaporthe* are widely distributed and have a broad range of hosts including economically significant agricultural crops and ornamental plants such as species of *Camellia*, *Castanea*, *Citrus*, *Glycine*, *Helianthus*, *Juglans*, *Persea*, *Pyrus*, *Vaccinium*, *Vitis* and many more (van Rensburg et al. 2006; Santos and Phillips 2009; Crous et al. 2011a, b, 2016; Santos et al. 2011; Thompson et al. 2011; Grasso et al. 2012; Huang et al. 2013; Lombard et al. 2014; Gao et al. 2015, 2016, 2017; Udayanga et al. 2012, 2015; Guarnaccia et al. 2016; Dissanayake et al. 2017; Guarnaccia and Crous 2017; Fan et al. 2018; Senanayake et al. 2018; Guo et al. 2020). *Diaporthe* species have been reported as destructive plant pathogens, harmless endophytes or saprobes (Murali et al. 2006; Udayanga et al. 2012; Gomes et al. 2013; Ménard et al. 2014; Guarnaccia et al. 2016; Torres et al. 2016; Senanayake et al. 2018). However, the biology and lifestyle of some of these fungi remain unclear (Vilka and Volkova 2015).

In the past, methods of species identification of *Diaporthe* had previously been based only on host as well as morphological characters such as the size and shape of ascomata and conidiomata. Nowadays, molecular phylogenetic studies demonstrate that determining species boundaries only by morphological characters is not possible due to lack of host specificity and their variability under changing environmental conditions (Gomes et al. 2013). Phylogenetic analysis using a five-locus dataset (ITS-TUB-TEF-CAL-HIS) has been determined to be the optimal combination to identify species of *Diaporthe* species, as revealed by Santos et al. (2017). Many *Diaporthe* species are described based on a polyphasic approach together with morphological characterisation (Rehner and Uecker 1994; Udayanga et al. 2011; Gao et al. 2017; Guarnaccia and Crous 2017; Yang et al. 2018a, 2020; Crous et al. 2020; Dayarathne et al. 2020; Guo et al. 2020; Hyde et al. 2020; Li et al. 2020; Zapata et al. 2020).

The aim of this study was to explore the diversity of *Diaporthe* species from symptomatic leaves of plants in Yunnan Province. We present three novel species and five known species of *Diaporthe*, collected from species belonging to ten host genera, based on morphological characters and phylogenetic analysis.

## Materials and methods

### Isolation and morphological studies

Leaves of samples were collected in Yunnan Province, China. Isolations from surface sterilized leaf tissues were conducted following the protocol of Gao et al. (2014). Tissue fragments (5 × 5 mm) were taken from the margin of leaf lesions and surface-sterilized by immersing them in 75% ethanol solution for 1 min, 5% sodium hypochlorite solution for 30 s, and then rinsing in sterile distilled water for 1 min. The pieces were dried with sterilized paper towels and placed on potato dextrose agar (PDA) (Cai et al. 2009). PDA plates (90 mm) were incubated in an incubator at 25 °C for 2–4 days, and hyphae were picked out of the periphery of the colonies and inoculated onto new PDA plates.

Following 2–3 weeks of incubation, photographs of colonies were taken at 7 days and 15 days using a Powershot G7X mark II digital camera. Colour notations was done using the colour charts of Rayner (1970). Micromorphological characters were observed using an Olympus SZX10 stereomicroscope and Olympus BX53 microscope, both fitted with Olympus DP80 high definition colour digital cameras to document fungal structures. All fungal strains were stored in 10% sterilized glycerin at 4 °C for further studies. Voucher and type specimens were deposited in the Herbarium of Plant Pathology, Shandong Agricultural University (HSAUP). Living cultures were deposited in the Shandong Agricultural University Culture Collection (SAUCC). Taxonomic information of the new taxa was submitted to MycoBank (http://www.mycobank.org).

### DNA extraction and amplification

Genomic DNA was extracted from fungal mycelium on PDA, using a modified cetyltrimethylammonium bromide (CTAB) protocol as described in Guo et al. (2000). The internal transcribed spacer regions with intervening 5.8S nrRNA gene (ITS), part of the beta-tubulin gene region (TUB), partial translation elongation factor 1-alpha (TEF), histone H3 (HIS) and calmodulin (CAL) genes were amplified and sequenced by using primers pairs ITS4/ITS5 (White et al. 1990), Bt2a/Bt2b (Glass and Donaldson 1995), EF1-728F/EF1-986R (Carbone and Kohn 1999), CAL-228F/CAL-737R (Carbone and Kohn 1999) and CYLH3F/H3-1b (Glass and Donaldson 1995; Crous et al. 2004), respectively.

PCR was performed using an Eppendorf Master Thermocycler (Hamburg, Germany). Amplification reactions were performed in a 25 μL reaction volume, which contained 12.5 μL Green Taq Mix (Vazyme, Nanjing, China), 1 μL of each forward and reverse primer (10 μM) (Biosune, Shanghai, China), and 1 μL template genomic DNA in amplifier, and were adjusted with distilled deionized water to a total volume of 25 μL.

PCR parameters were as follows: 95 °C for 5 min, followed by 35 cycles of denaturation at 95 °C for 30 s, annealing at a suitable temperature for 30 s, extension at 72 °C for 1 min and a final elongation step at 72 °C for 10 min. Annealing temperature for each gene were 55 °C for ITS, 60 °C for TUB, 52 °C for TEF, 54 °C for CAL and 57 °C for HIS. The PCR products were visualised on 1% agarose electrophoresis gel. Sequencing was done bi-directionally, conducted by the Biosune Company Limited (Shanghai, China). Consensus sequences were obtained using MEGA 7.0 (Kumar et al. 2016). All sequences generated in this study were deposited in GenBank (Table 1).

**Table 1.** Species and Genbank accession numbers of DNA sequences used in this study. New sequences in bold.

Species	Voucher	Host/Substrare	GeneBank accession number					Reference
			ITS	TUB	TEF	CAL	HIS	
*Diaporthe acuta*	PSCG 046	*Pyrus pyrifolia*	MK626958	MK691224	MK654803	MK691124	MK726162	Guo et al. 2020
	PSCG 047*	*Pyrus pyrifolia*	MK626957	MK691225	MK654802	MK691125	MK726161	Guo et al. 2020
*D. acutispora*	LC6160	*Camellia sasanqua*	KX986763	KX999194	KX999154	KX999273	KX999234	Gao et al. 2017
	LC6161	*Coffea* sp.	KX986764	KX999195	KX999155	KX999274	KX999235	Gao et al. 2017
*D. amaranthophila*	MAFF 246900	*Amaranthus tricolor*	LC459575	LC459579	LC459577	LC459583	LC459581	Rossman et al. 2015
	MAFF 246901	*Amaranthus tricolor*	LC459576	LC459580	LC459578	LC459584	LC459582	Rossman et al. 2015
*D. angelicae*	CBS 111592*	*Heracleum sphondylium*	KC343027	KC343995	KC343753	KC343269	KC343511	Gomes et al. 2013
*D. anhuiensis*	CNUCC 201901*	*Cunninghamia lanceolata*	MN219718	MN227008	MN224668	MN224549	MN224556	Zhou and Hou 2019
	CNUCC 201902	*Cunninghamia lanceolata*	MN219727	MN227009	MN224669	MN224550	MN224557	Zhou and Hou 2019
*D. arctii*	DP0482	*Arctium* sp.	KJ590736	KJ610891	KJ590776	KJ612133	KJ659218	Udayanga et al. 2015
*D. arecae*	CBS 161.64*	*Areca catechu*	KC343032	KC344000	KC343758	KC343274	KC343516	Gomes et al. 2013
	CBS 535.75	*Citrus* sp.	KC343033	KC344001	KC343759	KC343275	KC343517	Gomes et al. 2013
	**SAUCC194.18**	***Persea americana***	**MT822546**	**MT855743**	**MT855860**	**MT855631**	**MT855515**	**This study**
*D. arengae*	CBS 114979*	*Arenga engleri*	KC343034	KC344002	KC343760	KC343276	KC343518	Gomes et al. 2013
*D. aseana*	MFLUCC 12-0299a*	On dead leaves	KT459414	KT459432	KT459448	KT459464	–	Dissanayake et al. 2017
*D. beilharziae*	BRIP 54792*	*Indigofera australis*	JX862529	KF170921	JX862535	–	–	Tan et al. 2013
*D. biconispora*	ZJUD 60	*Citrus sinensis*	KJ490595	KJ490416	KJ490474	–	KJ490537	Huang et al. 2017
	ZJUD 61	*Fortunella margarita*	KJ490596	KJ490417	KJ490475	–	KJ490538	Huang et al. 2017
	ZJUD 62	*Citrus grandis*	KJ490597	KJ490418	KJ490476	–	KJ490539	Huang et al. 2017
*D. brasiliensis*	CBS 133183*	*Aspidosperma tomentosus*	KC343042	KC344010	KC343768	KC343284	KC343526	Gomes et al. 2013
*D. caatingaensis*	URM 7486*	*Tacinga inamoena*	KY085926	KY115600	KY115603	KY115597	KY115605	Crous et al. 2017
*D. camporesii*	JZB320143	*Urtica dioidca*	MN535309	MN561316	MN984254	–	–	Hyde et al. 2020
*D. caricae-papayae*	NIBM-ABIJP	*Carica papaya*	MN335224	–	–	–	–	Rossman et al. 2015
*D. caryae*	CFCC 52563	*Carya illinoensis*	MH121498	MH121580	MH121540	MH121422	MH121458	Yang et al. 2018
	CFCC 52564	*Carya illinoensis*	MH121499	MH121581	MH121541	MH121423	MH121459	Yang et al. 2018
*D. cercidis*	CFCC 52565	*Cercis chinensis*	MH121500	MH121582	MH121542	MH121424	MH121460	Yang et al. 2018
***D. chrysalidocarpi***	**SAUCC194.33**	***Chrysalidocarpus lutescens***	**MT822561**	**MT855758**	**MT855874**	**MT855645**	**MT855530**	**This study**
	SAUCC194.35*	***Chrysalidocarpus lutescens***	**MT822563**	**MT855760**	**MT855876**	**MT855646**	**MT855532**	**This study**
*D. cichorii*	MFLUCC 17-1023*	*Cichorium intybus*	KY964220	KY964104	KY964176	KY964133	–	Dissanayake et al. 2017
*D. compacta*	LC3083*	*Camellia sinensis*	KP267854	KP293434	KP267928	–	KP293508	Gao et al. 2016
*D. cucurbitae*	CBS 136.25	*Cucumis sativus*	KC343031	KC343999	KC343757	KC343273	KC343515	Udayanga et al. 2014
*D. cuppatea*	CBS 117499	*Aspalathus linearis*	KC343057	KC344025	KC343783	KC343299	KC343541	Udayanga et al. 2012
*D. decedens*	CBS 109772	*Corylus avellana*	KC343059	KC344027	KC343785	KC343301	KC343543	Gomes et al. 2013
*D. eugeniae*	CBS 444.82	*Eugenia aromatica*	KC343098	KC344066	KC343824	KC343340	KC343582	Gomes et al. 2013
*D. fraxini-angustifoliae*	BRIP 54781*	*Fraxinus angustifolius*	JX862528	KF170920	JX862534	–	–	Tan et al. 2013
*D. fulvicolor*	PSCG 051*	*Pyrus pyrifolia*	MK626859	MK691236	MK654806	MK691132	MK726163	Guo et al. 2020
	PSCG 057	*Pyrus pyrifolia*	MK626858	MK691233	MK654810	MK691131	MK726164	Guo et al. 2020
*D. ganjae*	CBS 180.91*	*Cannabis sativa*	KC343112	KC344080	KC343838	KC343354	KC343596	Gomes et al. 2013
*D. guangxiensis*	JZBH 320094*	*Vitis vinifera*	MK335772	MK500168	MK523566	MK736727	–	Manawasinghe et al. 2019
*D. gulyae*	MF-Ha 17-042*	*Helianthus annuus*	MK024252	MK033488	MK039420	–	–	Thompson et al. 2011
*D. hongkongensis*	CBS 115448*	*Dichroa febrifuga*	KC343119	KC344087	KC343845	KC343361	KC343603	Gomes et al. 2013
	CGMCC 3.17102	*Lithocarpus glaber*	KF576275	KF576299	KF576250	KF576227	–	Gao et al. 2015
	LC 3478	*Camellia sinensis*	KP267904	KP293484	KP267978	–	KP293553	Gao et al. 2017
	**SAUCC194.81**	***Millettia reticulata***	**MT822609**	**MT855806**	**MT855921**	**MT855688**	**MT855577**	**This study**
	**SAUCC194.87**	***Camellia sinensis***	**MT822615**	**MT855812**	**MT855927**	**MT855694**	**MT855583**	**This study**
*D. huangshanensis*	CNUCC 201903	*Camellia oleifera*	MN219729	MN227010	MN224670	–	MN224558	Zhou and Hou 2019
	CNUCC 201904	*Camellia oleifera*	MN219730	MN227011	MN224671	–	MN224559	Zhou and Hou 2019
*D. infecunda*	CBS 133812*	*Schinus terebinthifolius*	KC343126	KC344094	KC343852	KC343368	KC343610	Gomes et al. 2013
*D. krabiensis*	MFLUCC 17-2481*	*Bruguiera* sp.	MN047101	MN431495	MN433215	–	–	Dayarathne et al. 2020
*D. litchiicola*	BRIP 54900*	*Litchi chinensis*	JX862533	KF170925	JX862539	–	–	Tan et al. 2013
*D. limonicola*	CPC 28200*	*Citrus limon*	MF418422	MF418582	MF418501	MF418256	MF418342	Guarnaccia and Crous 2017
*D. lusitanicae*	CBS 123212*	*Foeniculum vulgare*	KC343136	KC344104	KC343862	KC343378	KC343620	Phillips and Santos 2009
***D. machili***	**SAUCC194.69**	***Pometia pinnata***	**MT822597**	**MT855794**	**MT855909**	**MT855677**	**MT855565**	**This study**
	SAUCC194.111*	***Machilus pingii***	**MT822639**	**MT855836**	**MT855951**	**MT855718**	**MT855606**	**This study**
*D. malorum*	CAA752*	*Malus domestica*	KY435643	KY435671	KY435630	KY435661	KY435651	Santos et al. 2017
	CAA740	*Malus domestica*	KY435642	KY435670	KY435629	KY435660	KY435650	Santos et al. 2017
*D. manihotia*	CBS 505.76	*Manihot utilissima*	KC343138	KC344106	KC343864	KC343380	KC343622	Gomes et al. 2013
*D. mayteni*	CBS 133185*	*Maytenus ilicicolia*	KC343139	KC344107	KC343865	KC343381	KC343623	Gomes et al. 2013
*D. melitensis*	CPC 27873*	*Citrus limon*	MF418424	MF418584	MF418503	MF418258	MF418344	Guarnaccia and Crous 2017
*D. middletonii*	BRIP 54884e*	*Rapistrum rugostrum*	KJ197286	KJ197266	KJ197248	–	–	Thompson et al. 2015
	**SAUCC194.27**	***Litchi chinensis***	**MT822555**	**MT855752**	**MT855868**	**MT855639**	**MT855524**	**This study**
	**SAUCC194.45**	***Lithocarpus glaber***	**MT822573**	**MT855770**	**MT855886**	**MT855654**	**MT855542**	**This study**
	**SAUCC194.46**	***Lithocarpus glaber***	**MT822574**	**MT855771**	**MT855887**	**MT855655**	**MT855543**	**This study**
	**SAUCC194.48**	***Lithocarpus craibianus***	**MT822576**	**MT855773**	**MT855889**	**MT855657**	**MT855545**	**This study**
*D. millettiae*	GUCC9167*	*Millettia reticulata*	MK398674	MK502089	MK480609	MK502086	–	Long et al. 2019
*D. multigutullata*	ZJUD 98*	*Citrus grandis*	KJ490633	KJ490454	KJ490512	–	KJ490575	Huang et al. 2015
*D. musigena*	CBS 129519*	*Musa* sp.	KC343143	KC344111	KC343869	KC343385	KC343627	Crous et al. 2011
*D. myracrodruonis*	URM7972	*Myracrodruon urundeuva*	MK205289	MK205291	MK213408	MK205290	–	Silva et al. 2019
*D. neoarctii*	CBS 109490*	*Ambrosia trifida*	KC343145	KC344113	KC343871	KC343387	KC343629	Gomes et al. 2013
*D. novem*	CBS 127270*	*Glycine max*	KC343156	KC344124	KC343882	KC343398	KC343640	Santos et al. 2011
*D. osmanthi*	GUCC9165*	*Osmanthus fragrans*	MK398675	MK502091	MK480610	MK502087	–	Long et al. 2019
	**SAUCC194.21**	***Litchi chinensis***	**MT822549**	**MT855746**	**MT855862**	**MT855634**	**MT855518**	**This study**
*D. oxe*	CBS 133186*	*Maytenus ilicifolia*	KC343164	KC344132	KC343890	KC343406	KC343648	Gomes et al. 2013
	CBS 133187	*Maytenus ilicifolia*	KC343165	KC344133	KC343891	KC343407	KC343649	Gomes et al. 2013
*D. pandanicola*	MFLUCC 17-0607	*Pandanus* sp.	MG646974	MG646930	–	–	–	Tibpromma et al. 2018
	**SAUCC194.82**	***Millettia reticulata***	**MT822610**	**MT855807**	**MT855922**	**MT855689**	**MT855578**	**This study**
*D. paranensis*	CBS 133184*	*Maytenus ilicifolia*	KC343171	KC344139	KC343897	KC343413	KC343655	Gomes et al. 2013
*D. pascoei*	BRIP 54847*	*Persea americana*	JX862532	KF170924	JX862538	–	–	Tan et al. 2013
*D. perseae*	CBS 151.73	*Persea gratissima*	KC343173	KC344141	KC343899	KC343415	KC343657	Gomes et al. 2013
*D. pescicola*	MFLU 16-0105*	*Prunus persica*	KU557555	KU557579	KU557623	KU557603	–	Dissanayake et al. 2017
*D. podocarpi-macrophylli*	LC6155*	*Podocarpus macrophyllus*	KX986774	KX999207	KX999167	KX999278	KX999246	Gao et al. 2017
	LC6200	*Podocarpus macrophyllus*	KX986769	KX999201	KX999161	KX999276	KX999240	Gao et al. 2017
***D. pometiae***	**SAUCC194.19**	***Persea americana***	**MT822547**	**MT855744**	**MT855861**	**MT855632**	**MT855516**	**This study**
	SAUCC194.72*	***Pometia pinnata***	**MT822600**	**MT855797**	**MT855912**	**MT855679**	**MT855568**	**This study**
	**SAUCC194.73**	***Heliconia metallica***	**MT822601**	**MT855798**	**MT855913**	**MT855680**	**MT855569**	**This study**
*D. pseudomangiferae*	CBS 101339*	*Mangifera indica*	KC343181	KC344149	KC343907	KC343423	KC343665	Gomes et al. 2013
*D. pseudophoenicicola*	CBS 462.69*	*Phoenix dactylifera*	KC343184	KC344152	KC343910	KC343426	KC343668	Gomes et al. 2013
*D. pterocarpicola*	MFLUCC 10-0580a*	*Pterocarpus indicus*	JQ619887	JX275441	JX275403	JX197433	–	Udayanga et al. 2012
	MFLUCC 10-0580b	*Pterocarpus indicus*	JQ619888	JX275442	JX275404	JX197434	–	Udayanga et al. 2012
*D. pyracanthae*	CAA487*	*Pyracantha coccinea*	KY435636	KY435667	KY435626	KY435657	KY435647	Santos et al. 2017
*D. racemosae*	CPC 26646*	*Euclea racemosa*	MG600223	MG600227	MG600225	MG600219	MG600221	Marin-Felix et al. 2018
*D. raonikayaporum*	CBS 133182*	*Spondias mombin*	KC343188	KC344156	KC343914	KC343430	KC343672	Gomes et al. 2013
*D. rossmaniae*	CAA 762*	*Vaccinium corymbosum*	MK792290	MK837914	MK828063	MK883822	MK871432	Hilario et al. 2020
*D. sackstonii*	BRIP 54669b*	*Helianthus annuus*	KJ197287	KJ197267	KJ197249	–	–	Thompson et al. 2015
*D. salinicola*	MFLU 18-0553*	*Xylocarpus* sp.	MN047098	–	MN077073	–	–	Dayarathne et al. 2020
	MFLU 17-2592	*Xylocarpus* sp.	MN047099	–	MN077074	–	–	Dayarathne et al. 2020
*D. schini*	CBS 133181*	*Schinus terebinthifolius*	KC343191	KC344159	KC343917	KC343433	KC343675	Gomes et al. 2013
*D. schoeni*	MFLU 15-2609	*Schoenus nigricans*	KY964229	KY964112	KY964185	KY964141	–	Dissanayake et al. 2017
*D. sennae*	CFCC 51636*	*Senna bicapsularis*	KY203724	KY228891	KY228885	KY228875	–	Yang et al. 2017
*D. serafiniae*	BRIP 55665a*	*Helianthus annuus*	KJ197274	KJ197254	KJ197236	–	–	Thompson et al. 2015
*D. spinosa*	PSCG 383*	*Pyrus pyrifolia*	MK626849	MK691234	MK654811	MK691129	MK726156	Guo et al. 2020
*D. stewartii*	CBS 193.36*	*Cosmos bipinnatus*	FJ889448	JX275421	GQ250324	JX197415	–	Santos et al. 2010; Udayanga et al. 2012
*D. subordinaria*	CBS 101711	*Plantago lanceolata*	KC343213	KC344181	KC343939	KC343455	KC343697	Gomes et al. 2013
	CBS 464.90	*Plantago lanceolata*	KC343214	KC344182	KC343940	KC343456	KC343698	Gomes et al. 2013
*D. taoicola*	PSGG485	*Prunus persica*	MK626869	MK691227	MK654812	MK691120	MK726173	Dissanayake et al. 2017
*D. tarchonanthi*	CPC 37479	*Tarchonanthus littoralis*	MT223794	–	–	–	–	Crous et al. 2020
*D. tectonigena*	MFLUCC 12-0767*	*Tectona grandis*	KU712429	KU743976	KU749371	KU749358	–	Doilom et al. 2016
*D. terebinthifolii*	CBS 133180*	*Schinus terebinthifolius*	KC343216	KC344184	KC343942	KC343458	KC343700	Gomes et al. 2013
*D. undulate*	LC6624*	Unknown host	KX986798	KX999230	KX999190	–	KX999269	Gao et al. 2017
	LC8110	Unknown host	KY491545	KY491565	KY491555	–	–	Gao et al. 2017
*D. vawdreyi*	BRIP 57887a*	*Psidium guajava*	KR936126	KR936128	KR936129	–	–	Crous et al. 2015
*D. viniferae*	JZBH 320071	*Vitis vinifera*	MK341550	MK500112	MK500107	MK500119	–	Manawasinghe et al. 2019
	JZBH 320072	*Vitis vinifera*	MK341551	MK500113	MK500108	MK500120	–	Manawasinghe et al. 2019
*D. xishuangbanica*	LC6707*	*Camellia sinensis*	KX986783	KX999216	KX999175	–	KX999255	Gao et al. 2017
*Diaporthella corylina*	CBS 121124	*Corylus* sp.	KC343004	KC343972	KC343730	KC343246	KC343488	Gomes et al. 2013

Isolates marked with “*” are ex-type or ex-epitype strains.

### Phylogenetic analyses

Novel sequences generated from the sixteen strains in this study, and all reference sequences of *Diaporthe* species downloaded from GenBank, were used for phylogenetic analyses. Alignments of the individual locus were determined using MAFFT v. 7.110 by default settings (Katoh et al. 2017) and manually corrected where necessary. To establish the identity of the isolates at species level, phylogenetic analyses were conducted first individually for each locus and then as combined analyses of five loci (ITS, TUB, TEF, CAL and HIS regions). Phylogenetic analyses were based on maximum likelihood (ML) and Bayesian inference (BI) for the multi-locus analyses. For BI, the best evolutionary model for each partition was determined using MrModeltest v. 2.3 (Nylander 2004) and incorporated into the analyses. ML and BI were run on the CIPRES Science Gateway portal (https://www.phylo.org/) (Miller et al. 2012) using RaxML-HPC2 on XSEDE (8.2.12) (Stamatakis 2014) and MrBayes on XSEDE (3.2.7a) (Huelsenbeck and Ronquist 2001; Ronquist and Huelsenbeck 2003; Ronquist et al. 2012), respectively. For ML analyses the default parameters were used and BI was carried out using the rapid bootstrapping algorithm with the automatic halt option. Bayesian analyses included five parallel runs of 5,000,000 generations, with the stop rule option and a sampling frequency of 500 generations. The burn-in fraction was set to 0.25 and posterior probabilities (PP) were determined from the remaining trees. The resulting trees were plotted using FigTree v. 1.4.2 (http://tree.bio.ed.ac.uk/software/figtree) and edited with Adobe Illustrator CS5.1. New sequences generated in this study were deposited at GenBank (https://www.ncbi.nlm.nih.gov; Table 1) and the alignments and trees were deposited in TreeBASE: S27479 (http://treebase.org/treebase-web/home.html).

## Results

### Phylogenetic analyses

Sixteen strains of *Diaporthe* isolated from plant hosts from Yunnan, China, were grown in culture and used for analyses of molecular sequence data. *Diaporthe* spp. were analysed by using multilocus data (ITS, TUB, TEF, CAL and HIS) from 115 isolates of *Diaporthe* spp. and *Diaporthella corylina* (CBS 121124) as the outgroup taxon. A total of 3005 characters including gaps were obtained in the phylogenetic analysis, viz. ITS: 1–656, TUB: 657–1329, TEF: 1330–1860, CAL: 1861–2444, HIS: 2445–3005. Of these characters, 1349 were constant, 453 were variable and parsimony-uninformative, and 1203 were parsimony-informative. For the BI and ML analyses, the substitution model GTR+I+G for ITS, TUB, TEF and HIS, HKY+I+G for and CAL were selected and incorporated into the analyses. The ML tree topology confirmed the tree topologies obtained from the BI analyses, and therefore, only the ML tree is presented (Fig. 1).

**Figure 1.**
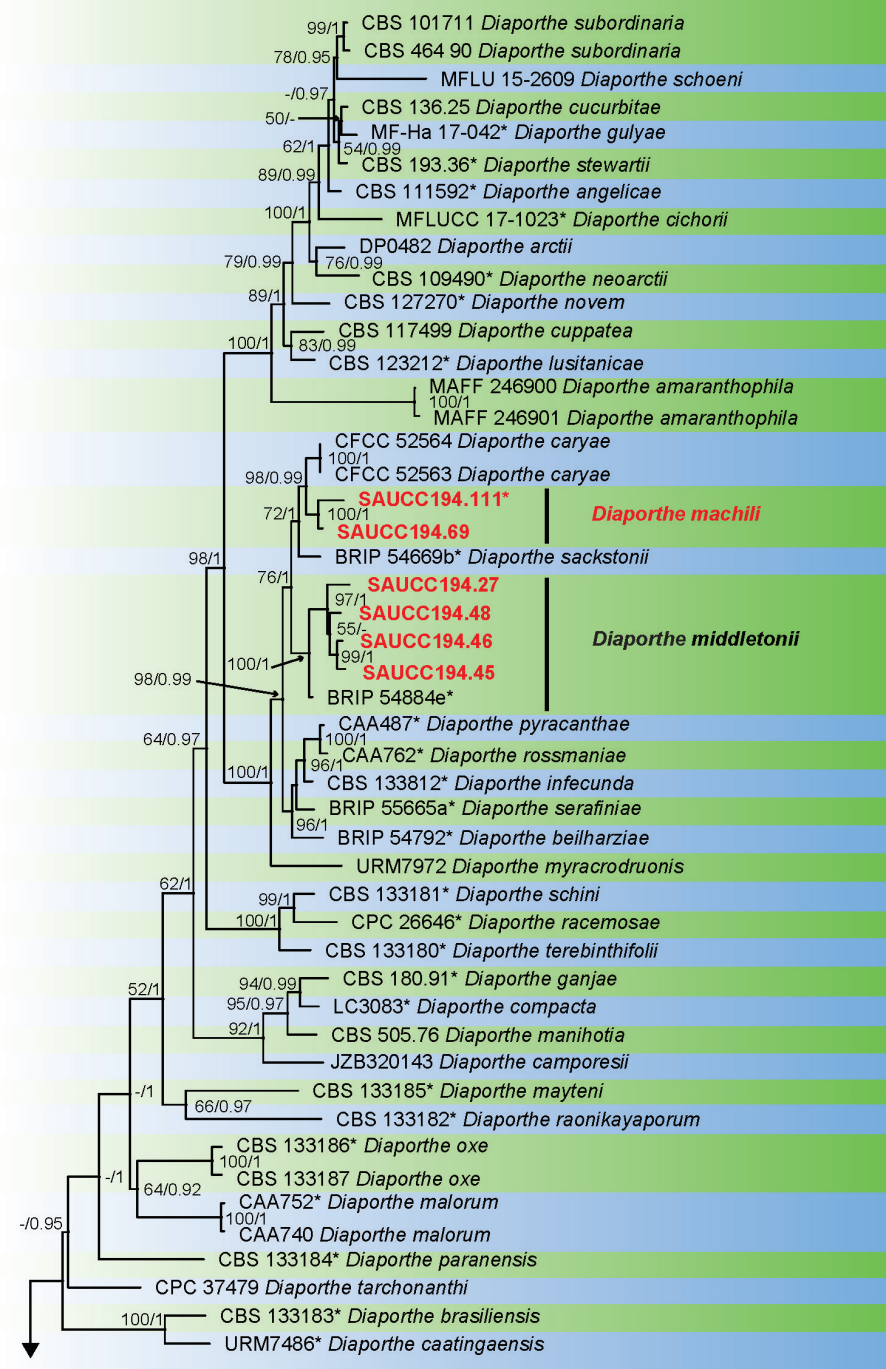
Phylogram of *Diaporthe* spp. based on combined sequence data of ITS, TUB, TEF, CAL and HIS genes. The ML and BI bootstrap support values above 50% and 0.90 BYPP are shown at the first and second position, respectively. Strains marked with “*” are ex-type or ex-epitype. Codes referring to strains from the current study are written in red. Some branches were shortened to fit them to the page as indicated by two diagonal lines with the number of times a branch was shortened indicated.

**Figure 1.**
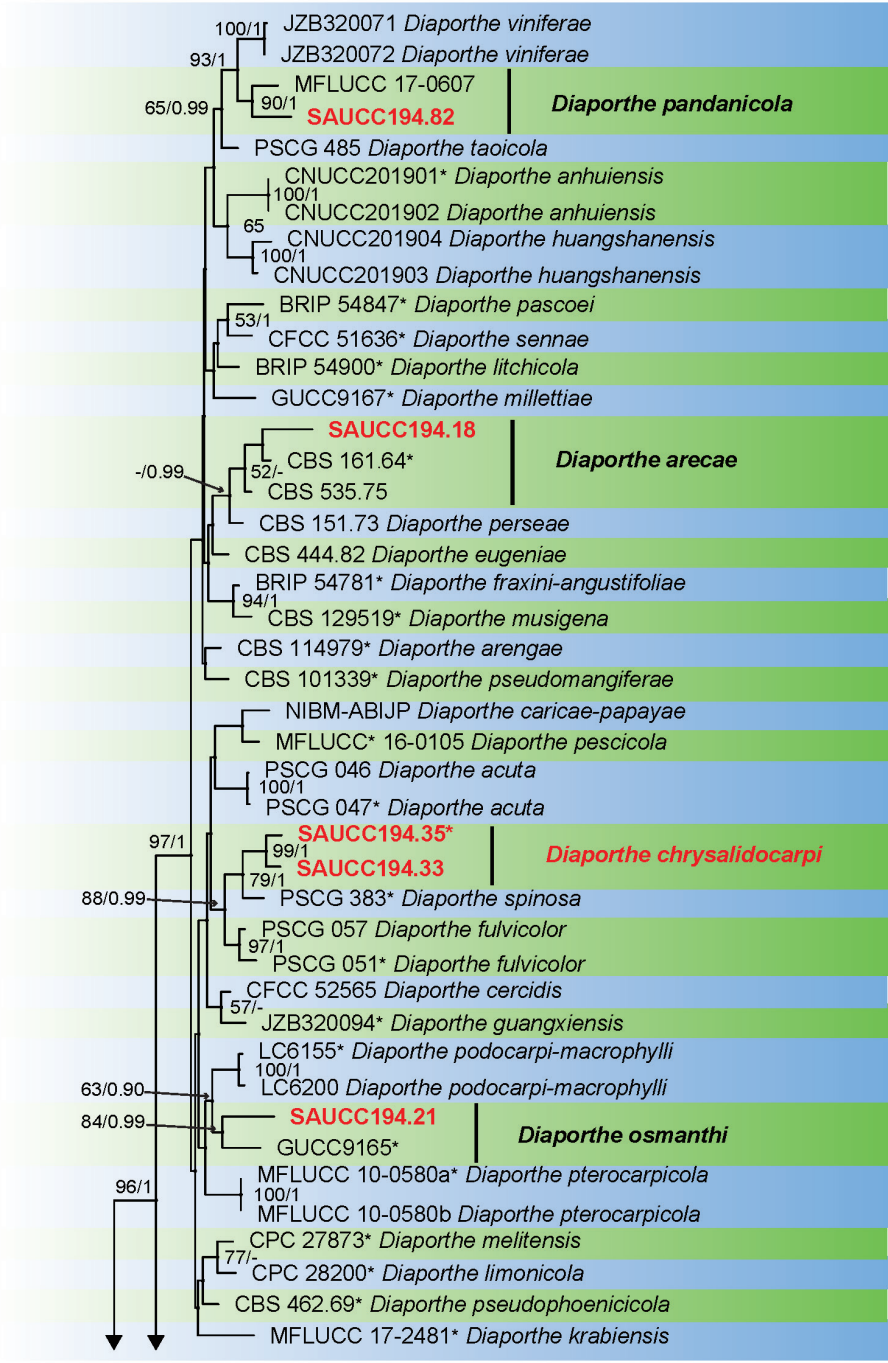
Continued.

**Figure 1.**
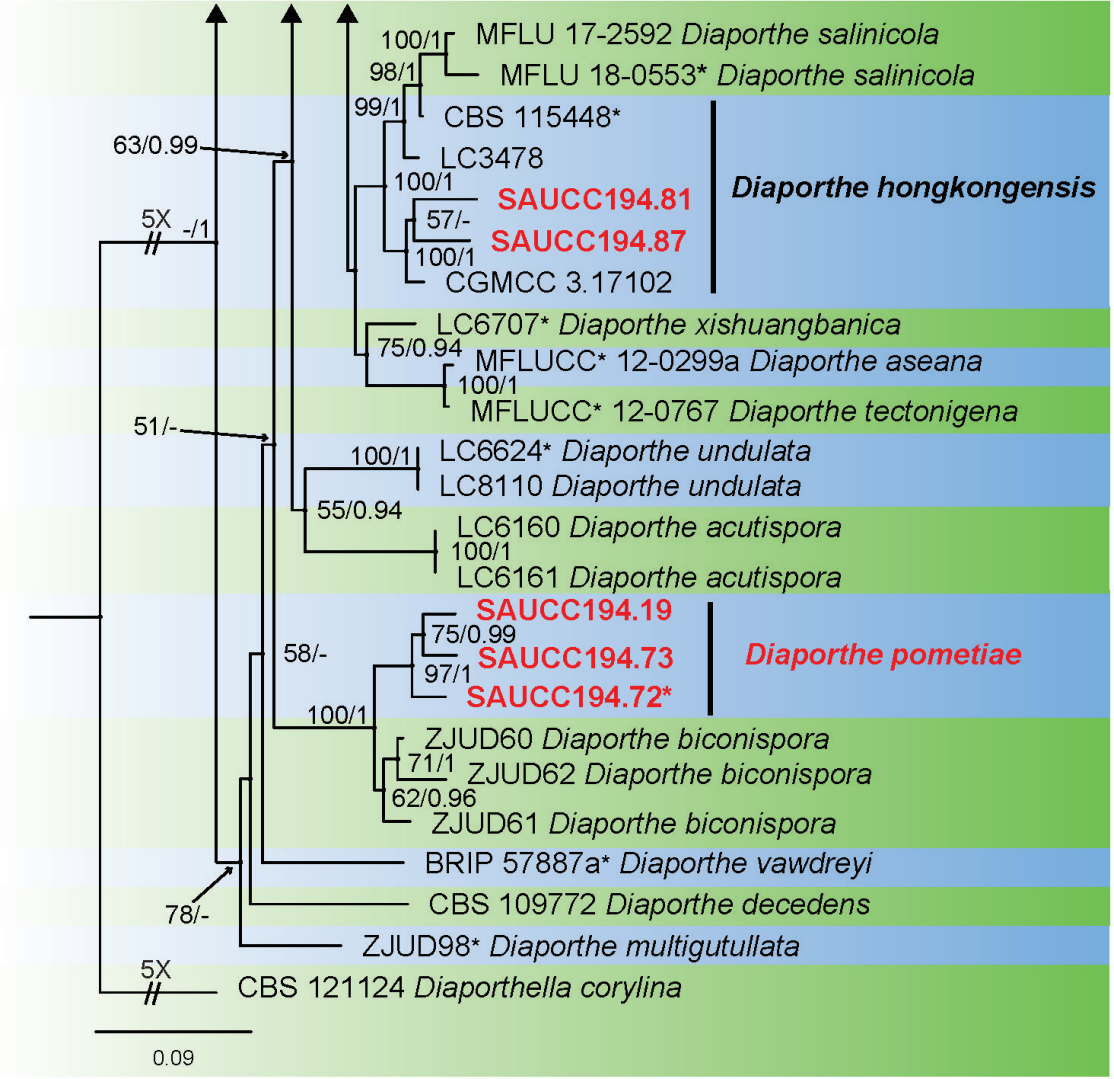
Continued.

ML bootstrap support values (≥ 50%) and Bayesian posterior probability (≥ 0.90) are shown as first and second position above nodes, respectively. Based on the five-locus phylogeny and morphology, nine isolates were assigned to five species, including *Diaporthe arecae* (1), *D. hongkongensis* (2), *D. middletonii* (4), *D. osmanthi* (1) and *D. pandanicola* (1), whereas seven isolates formed distinct well supported clades, which refer to novel species named *D. chrysalidocarpi* (2), *D. machili* (2) and *D. pometiae* (3), respectively.

### Taxonomy

#### Diaporthe arecae

Taxon classificationFungiDiaporthalesDiaporthaceae

(H.C. Srivast., Zakia & Govindar.) R.R. Gomes, Glienke & Crous, Persoonia 31: 16. (2013)

49466F7B-A01E-51FB-A01C-15E29B1B35D9

Figure 2

Subramanella arecae H.C. Srivast., Zakia & Govindar., in Srivastava, Banu and Govindarajan (1962). Basionym.

##### Description.

Asexual morph: Conidiomata pycnidial, several pycnidia grouped together, globose, black, erumpent, exuding creamy to yellowish conidial droplets from ostioles. Conidiophores hyaline, septate, branched, cylindrical, straight to sinuous, 25.0–32.0 × 1.4–2.5 μm. Conidiogenous cells 10.5–20.7 × 1.4–2.0 μm, phialidic, cylindrical, swollen at base, tapering towards apex, slightly curved. Alpha conidia hyaline, smooth, aseptate, ellipsoidal, guttulate, apex subobtuse, base subtruncate, 7.5–10.0 × 1.8–3.0 µm (mean = 8.2 × 2.4 μm, n = 20). Beta conidia hyaline, aseptate, filiform, slightly curved, tapering towards base, 18.5–26.5 × 1.0–1.8 µm (mean = 24.3 × 1.4 μm, n = 20). Gamma conidia not observed. Sexual morph not observed.

##### Culture characteristics.

Cultures incubated on PDA at 25 °C in darkness, growth rate 11.2–13.3 mm diam/day. Aerial mycelium white, cottony, feathery, abundant in center, sparse in margin, white on surface, reverse yellowish to tan.

##### Specimen examined.

China, Yunnan Province: Xishuangbanna Tropical Botanical Garden, Chinese Academy of Sciences, on diseased leaves of *Persea americana* (Lauraceae). 19 April 2019, S.T. Huang, HSAUP194.18, living culture SAUCC194.18.

**Figure 2.**
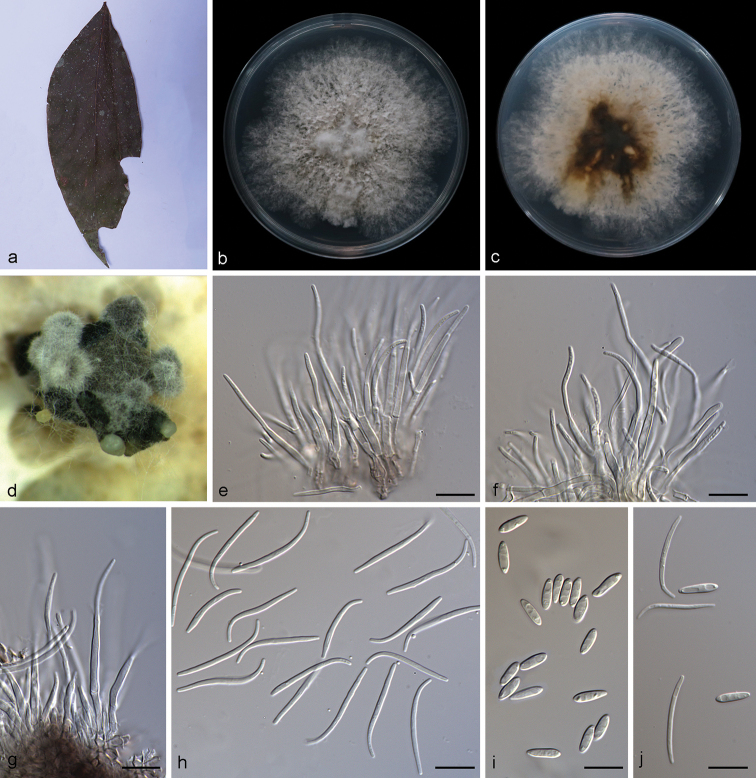
*Diaporthe arecae* (SAUCC194.18) **a** infected leaf of *Persea americana***b, c** surface and reverse of a colony after 15 days on PDA**d** conidiomata **e–g** conidiophores and conidiogenous cells **h** beta conidia **i** alpha conidia **j** alpha conidia and beta conidia. Scale bars: 10 μm (**e–j**).

##### Notes.

*Diaporthe arecae* (CBS 161.64) was originally described as *Subramanella arecae* on fruit of *Areca catechu* in India (Srivastava et al. 1962) and placed in *Diaporthe* by Gomes et al. (2013). The *Diaporthe* isolate from fruits of *Citrus* sp. (CBS 535.75) in Suriname was also placed in *D. arecae* by Gomes et al. (2013). In the present study, strain (SAUCC194.18) from symptomatic leaves of *Persea americana* was congruent with *D. arecae* based on morphology and DNA sequences data (Fig. 1). We therefore consider the isolated strain as *D. arecae*.

#### Diaporthe chrysalidocarpi

Taxon classificationFungiDiaporthalesDiaporthaceae

S.T. Huang, J.W. Xia, W.X. Sun, & X.G. Zhang sp. nov.

F02A5541-B8BA-55D2-92D9-190AD164B410

837812

Figure 3

##### Etymology.

Named after the host genus on which it was collected, *Chrysalidocarpus lutescens*.

##### Diagnosis.

*Diaporthe chrysalidocarpi* can be distinguished from the phylogenetically most closely related species *D. spinosa* by longer beta conidia (28.0–32.5 × 1.2–1.6 vs. 18.5–30.5 × 1.0–1.5 μm), and from other species *D. fulvicolor* by the types of conidia (*D. chrysalidocarpi* produces only beta conidia, while *D. fulvicolor* produces only alpha conidia) and several loci (25/491 in the ITS region, 18/471 TUB, 4/298 TEF, 28/458 CAL and 13/441 HIS).

##### Type.

China, Yunnan Province: Xishuangbanna Tropical Botanical Garden, Chinese Academy of Sciences, on diseased leaves of *Chrysalidocarpus lutescens* (Palmae). 19 April 2019, S.T. Huang, HSAUP194.35 holotype, ex-type living culture SAUCC194.35.

##### Description.

Asexual morph: Leaf spots irregular, pale brown in center, brown to tan at margin. Conidiomata pycnidial, scattered or aggregated, black, erumpent, raising above surface of culture medium, subglobose, exuding white or yellowish creamy conidial droplets from central ostioles after 30 days in light at 25 °C; pycnidial wall consists of black to dark brown, thin-walled cells. Conidiophores 27.5–35.0 × 1.4–2.0 μm, hyaline, slightly branched, swelling at base, subcylindrical, septate, smooth, straight or curved. Conidiogenous cells 10.5–23.0 × 1.4–1.8 μm, phialidic, cylindrical, terminal, straight to sinuous, tapering towards apex. Beta conidia 28.0–32.5 × 1.2–1.6 μm (mean = 30.3 × 1.3 μm, n = 20), filiform, hyaline, straight or slightly curved, aseptate, base subtruncate, tapering towards the base. Alpha conidia and gamma conidia not observed. Sexual morph not observed.

##### Culture characteristics.

Cultures incubated on PDA at 25 °C in darkness, growth rate 13.3–15.2 mm diam/day, initially white, becoming greyish, reverse pale brown, with concentric rings of dense, sparse hyphae, irregular margin, fluffy aerial mycelium at center, pycnidia forming after 15 days.

##### Additional specimen examined.

China, Yunnan Province: Xishuangbanna Tropical Botanical Garden, Chinese Academy of Sciences, on diseased leaves of *Chrysalidocarpus lutescens* (Palmae). 19 April 2019, S.T. Huang, HSAUP194.33 paratype; living culture SAUCC194.33.

**Figure 3.**
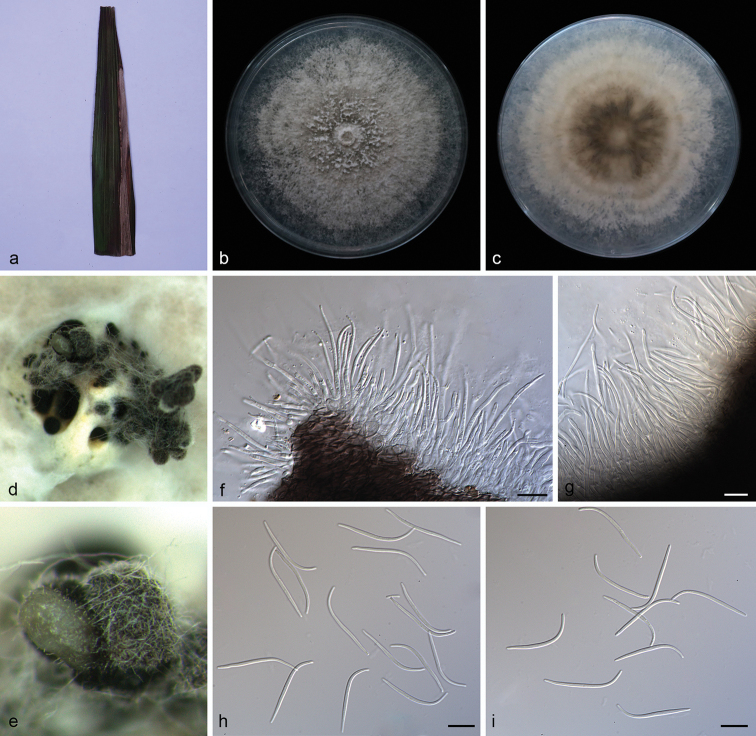
*Diaporthe chrysalidocarpi* (SAUCC194.35) **a** diseased leaf of *Chrysalidocarpus lutescens***b, c** surface and reverse of a colony after 15 days on PDA**d, e** conidiomata **f, g** conidiophores and conidiogenous cells **h, i** beta conidia. Scale bars: 10 μm (**f–i**).

##### Notes.

Phylogenetic analysis of a combined five gene showed that *D. chrysalidocarpi* formed an independent clade (Fig. 1) and is phylogenetically distinct from *D. spinosa* and *D. fulvicolor*. This species can be distinguished from *D. spinosa* by 61 different nucleotides in the concatenated alignment (13/492 in the ITS region, 17/471 TUB, 4/298 TEF, 17/458 CAL and 10/441 HIS), and *D. fulvicolor* by 88 nucleotides (25/491 in the ITS region, 18/471 TUB, 4/298 TEF, 28/458 CAL and 13/441 HIS). Morphologically, *D. chrysalidocarpi* differs from *D. spinosa* in having longer beta conidia (28.0–32.5 × 1.2–1.6 vs. 18.5–30.5 × 1.0–1.5 μm) (Guo et al. 2020). Furthermore, *Diaporthe chrysalidocarpi* produces only beta conidia, while *D. spinosa* produces alpha conidia and beta conidia and *D. fulvicolor* produces only alpha conidia (Guo et al. 2020). Therefore, we establish this fungus as a novel species.

#### Diaporthe hongkongensis

Taxon classificationFungiDiaporthalesDiaporthaceae

R.R. Gomes, Glienke, Crous, Persoonia 31: 23. (2013)

D074B02D-1506-5551-AB26-43FB98A81B31

Figure 4

##### Description.

Asexual morph: Conidiomata pycnidial, subglobose or globose, solitary, black, erumpent, coated with white hyphae, thick-walled, exuding creamy conidial droplets from central ostioles. Conidiophores hyaline, smooth, septate, unbranched, densely aggregated, cylindrical or clavate, straight to sinuous, swollen at base, tapering towards apex, 32.0–42.0 × 2.0–2.9 μm. Conidiogenous cells 20.0–24.2 × 1.3–2.3 μm, phialidic, cylindrical, terminal, slightly tapering towards apex. Alpha conidia, hyaline, smooth, aseptate, ellipsoidal or oval, 0–2 guttulate, apex subobtuse, base subtruncate, 5.5–7.0 × 2.0–2.5 µm (mean = 6.2 × 2.2 μm, n = 20). Beta conidia hyaline, aseptate, filiform, hamate, tapering towards both ends, mostly J-shaped, 21.5–27.0 × 1.4–1.8 µm (mean = 25.6 × 1.3 μm, n = 20). Gamma conidia not observed. Sexual morph not observed.

##### Culture characteristics.

Cultures incubated on PDA at 25 °C in darkness, growth rate 19.0–21.5 mm diam/day, cottony, radial with abundant aerial mycelium, sparse at margin, with an obvious pale brown concentric ring of dense hyphae, white to grayish on surface with age, white to pale brown on the reverse side.

**Figure 4.**
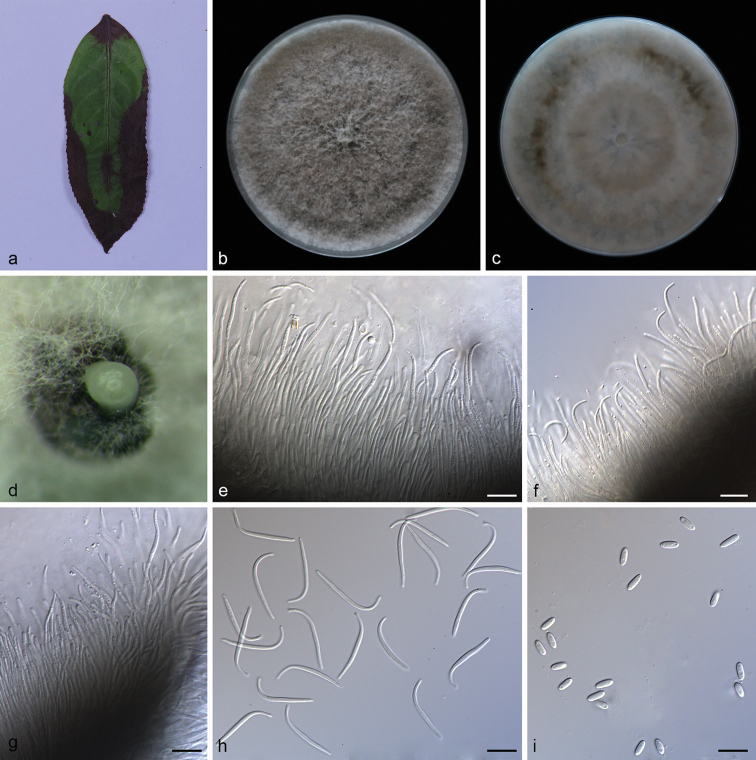
*Diaporthe hongkongensis* (SAUCC194.87) **a** diseased leaf of *Camellia sinensis***b, c** surface and reverse of colony after 15 days on PDA**d** conidiomata **e–g** conidiophores and conidiogenous cells **h** beta conidia **i** alpha conidia. Scale bars: 10 μm (**e–i**).

##### Specimens examined.

China, Yunnan Province: Xishuangbanna Tropical Botanical Garden, Chinese Academy of Sciences, 19 April 2019, S.T. Huang. On diseased leaves of *Millettia reticulata* (Fabaceae) HSAUP194.81, living culture SAUCC194.81; on diseased leaves of *Camellia sinensis* (Theaceae) HSAUP194.87, living culture SAUCC194.87.

##### Notes.

In the present study, two strains (SAUCC194.81 and SAUCC194.87) from symptomatic leaves of *Millettia reticulata* and *Camellia sinensis* were similar to *Diaporthe hongkongensis* (CGMCC 3.17102) (Gomes et al. 2013) and *D. salinicola* (MFLU 18-0553) (Dayarathne et al. 2020) based on DNA sequences data (Fig. 1). Morphologically, our strains were similar to *Diaporthe hongkongensis*, which was originally described with an asexual morph on fruits of *Dichroa febrifuga* in China, but the asexual morph of *D. salinicola* was undetermined. We therefore identify our strains as *D. hongkongensis*.

#### Diaporthe machili

Taxon classificationFungiDiaporthalesDiaporthaceae

S.T. Huang, J.W. Xia, W.X. Sun, & X.G. Zhang sp. nov.

06BE2C60-1305-5EA2-87B4-B6E27EA3E874

837814

Figure 5

##### Etymology.

Named after the host genus on which it was collected, *Machilus pingii*.

##### Diagnosis.

*Diaporthe machili* differs from *D. caryae* and *D. sackstonii* in the types of conidia (*D. machili* only produces beta conidia, while *D. caryae* produces alpha conidia and beta conidia, and *D. sackstonii* only produces alpha conidia), and from *D. caryae* in longer beta conidia (29.0–39.0 × 1.3–1.5 vs. 15.5–34.0 × 1.1–1.4 μm).

##### Type.

China, Yunnan Province: Xishuangbanna Tropical Botanical Garden, Chinese Academy of Sciences, on diseased leaves of *Machilus pingii* (Lauraceae). 19 April 2019, S.T. Huang, HSAUP194.111 holotype, ex-holotype living culture SAUCC194.111.

**Figure 5.**
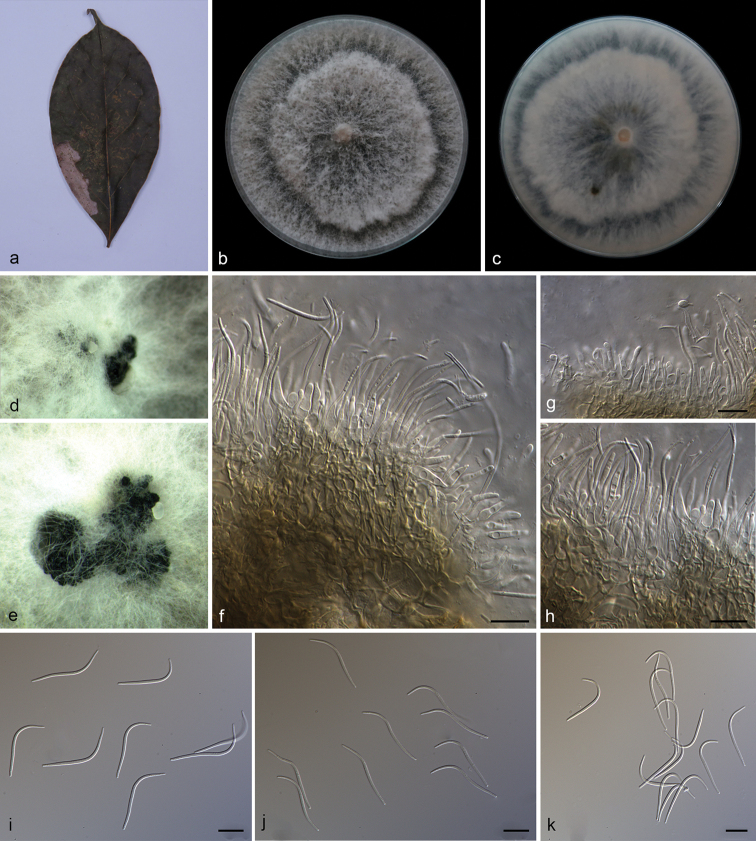
*Diaporthe machili* (SAUCC194.111) **a** infected leaf of *Machilus pingii***b, c** surface and reverse of colony after 15 days on PDA**d, e** conidiomata **f–h** conidiophores and conidiogenous cells **i–k** beta conidia. Scale bars: 10 μm (**f–k**).

##### Description.

Asexual morph: Conidiomata pycnidial, aggregated, black, erumpent, subglobose to globose, exuding creamy conidial droplets from central ostioles after 30 days in light at 25 °C. Conidiophores 7.0–11.4 × 1.8–2.8 μm, hyaline, unbranched, densely aggregated, mostly ampulliform, cylindrical, guttulate, septate, straight or slightly curved, swelling at base, tapering towards apex. Beta conidia 29.0–39.0 × 1.3–1.5 μm (mean = 32.5 × 1.4 μm, n = 20), filiform, hyaline, aseptate, mostly curved, J-shaped, swelling in middle, tapering towards both ends. Alpha and gamma conidia not observed. Sexual morph not observed.

##### Culture characteristics.

Cultures incubated on PDA at 25 °C in darkness, growth rate 16.3–17.5 mm diam/day, aerial mycelium abundant, white on surface, reverse white to pale yellow, with an obvious concentric zonation, pycnidia forming after 15 days.

##### Additional specimen examined.

China, Yunnan Province: Xishuangbanna Tropical Botanical Garden, Chinese Academy of Sciences, on diseased leaves of *Pometia pinnata* (Sapindaceae). 19 April 2019, S.T. Huang, HSAUP194. 69 paratype; living culture SAUCC194. 69.

##### Notes.

In the phylogenetic tree, *Diaporthe machili* forms an independent clade and is phylogenetically distinct from *D. caryae* and *D. sackstonii* (Fig. 1). *Diaporthe machili* can be distinguished from *D. caryae* in ITS, TUB, TEF, CAL and HIS loci by 67 nucleotide differences in concatenated alignment (5/459 in ITS, 10/416 in TUB, 15/334 in TEF, 7/454 in CAL and 30/455 in HIS), and from *D. sackstonii* in ITS, TUB and TEF loci by 58 nucleotide differences (12/559 in ITS, 23/486 in TUB and 23/348 in TEF). Moreover, *Diaporthe machili* differs from *D. caryae* in having longer beta conidia (29.0–39.0 × 1.3–1.5 vs. 15.5–34.0 × 1.1–1.4 μm). *Diaporthe machili* only produces beta conidia, while *D. caryae* produces alpha conidia and beta conidia, and *D. sackstonii* only produces alpha conidia (Thompson et al. 2015; Yang et al. 2018b).

#### Diaporthe middletonii

Taxon classificationFungiDiaporthalesDiaporthaceae

R.G. Shivas, L. Morin, S.M. Thomps. & Y.P. Tan, Persoonia 35: 45. (2015)

F77900E1-889B-5291-AEC8-995A46C92870

Figure 6

##### Description.

Asexual morph: Leaf spots discoid to irregular. Conidiomata pycnidial, scattered or aggregated in groups of 3–5 pycnidia, globose, black, erumpent, coated with white to greyish hyphae, thick-walled, exuding creamy translucent conidial droplets from central ostioles. Conidiophores hyaline, smooth, septate, unbranched, densely aggregated, cylindrical, straight to sinuous, tapering towards apex, 10.0–14.0 × 1.3–2.3 μm. Conidiogenous cells 5.0–9.5 × 1.3–1.7 μm, phialidic, cylindrical, terminal, slightly tapering towards apex. Alpha conidia hyaline, smooth, aseptate, biguttulate, ellipsoidal, oval, apex subobtuse, base subtruncate, 5.5–7.0 × 2.5–3.2 µm (mean = 6.3 × 2.8 μm, n = 20). Beta conidia hyaline, aseptate, filiform, mostly curved by 90–180°, tapering towards both ends, 26.0–36.5 × 1.0–1.6 µm (mean = 21.5 × 1.2 μm, n = 20). Gamma conidia not observed. Sexual morph not observed.

**Figure 6.**
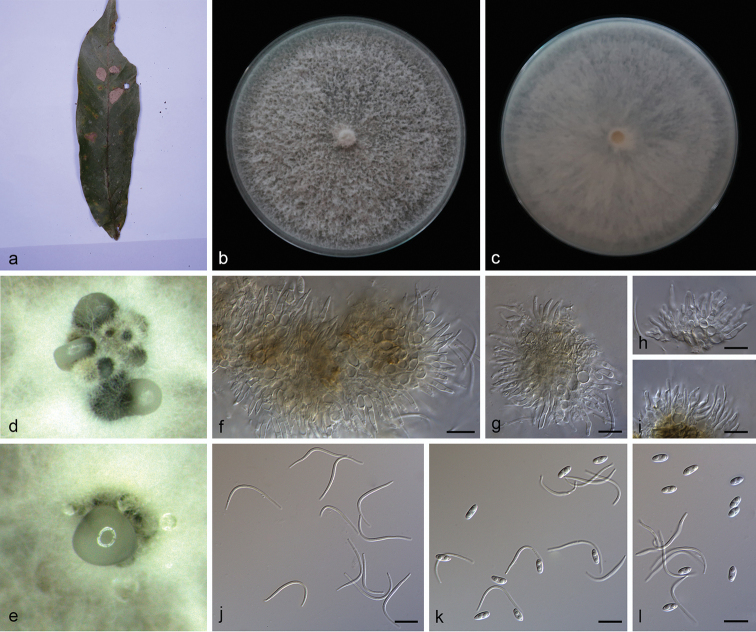
*Diaporthe middletonii* (SAUCC194.46) **a** infected leaf of *Lithocarpus glaber***b, c** surface and reverse of colony after 15 days on PDA**d, e** conidiomata **f–i** conidiophores and conidiogenous cells **j** beta conidia **k, l** alpha conidia and beta conidia. Scale bars: 10 μm (**f–l**).

##### Culture characteristics.

Cultures incubated on PDA at 25 °C in darkness, growth rate 22.5–24.0 mm diam/day, fluffy with abundant aerial mycelium, margin fimbriate, white on surface, white to pale yellow on reverse.

##### Specimens examined.

China, Yunnan Province: Xishuangbanna Tropical Botanical Garden, Chinese Academy of Sciences, 19 April 2019, S.T. Huang. On diseased leaves of *Litchi chinensis* (Sapindaceae), HSAUP194.27, living culture SAUCC194.27; on diseased leaves of *Lithocarpus glaber* (Fagaceae), HSAUP194.45, living culture SAUCC194.45; on diseased leaves of *Lithocarpus glaber* (Fagaceae), 19 April 2019, S.T. Huang, HSAUP194.46, living culture SAUCC194.46; on diseased leaves of *Lithocarpus craibianus* (Fagaceae), HSAUP194.48, living culture SAUCC194.48.

##### Notes.

*Diaporthe middletonii* was originally described from the stem of *Rapistrum rugosum* (BRIP 54884e) (Brassicaceae) and Chrysanthemoides monilifera subsp. rotundata (BRIP 57329) (Asteraceae) in Australia (Thompson et al. 2015). In the present study, four strains (SAUCC194.27, SAUCC194.45, SAUCC194.46 and SAUCC194.48) are closely related to *D. middletonii* in the combined phylogenetic tree (Fig. 1). The differences between nucleotides in the concatenated alignment (17/565 in ITS, 9/494 in TUB and 10/340 in TEF) were minor. Morphologically, our strains were similar to *D. middletonii* by slightly shorter and wider alpha conidia (5.0–7.0 × 2.5–3.2 vs. 6.0–7.5 × 2.0–2.5 μm), and longer beta conidia (26.0–36.5 × 1.0–1.6 vs. 20.0–35.0 × 1.0–1.5 μm) (Thompson et al. 2015). We therefore identify our strains as *Diaporthe middletonii*.

#### Diaporthe osmanthi

Taxon classificationFungiDiaporthalesDiaporthaceae

H. Long, K.D. Hyde, & Yong Wang bis, MycoKeys 57: 120. (2019)

B8C2511F-5EDA-5C24-A327-D4A794BFA96E

Figure 7

##### Description.

Conidiomata pycnidial, globose, 5–10 pycnidia grouped together, dark brown to black, exuding creamy to yellowish conidial droplets from central ostioles. Conidiophores hyaline, smooth, densely aggregated, branched, cylindric-clavate, 20.5–32.0 × 1.8–2.4 μm. Conidiogenous cells phialidic, hyaline, terminal, cylindrical, straight, 14.0–20.5 × 1.5–2.0 μm, tapered towards apex. Alpha conidia hyaline, aseptate, fusiform, tapering towards both ends, guttulate, 7.3–9.3 × 1.8–2.3 μm (mean = 8.5 × 2.0 μm, n = 20). Beta conidia hyaline, aseptate, filiform, curved, 22.0–28.5 × 1.0–2.0 μm (mean = 27.2 × 1.3 μm, n = 20). Gamma conidia not observed. Sexual morph not observed.

##### Culture characteristics.

Cultures incubated on PDA at 25 °C in darkness, growth rate 12.0–13.5 mm diam/day, cottony with abundant aerial mycelium, sparse at margin. With several concentric rings of dense hyphae, white on surface, white to pale brown on reverse.

##### Specimen examined.

China, Yunnan Province: Xishuangbanna Tropical Botanical Garden, Chinese Academy of Sciences, 19 April 2019, S.T. Huang. On diseased leaves of *Litchi chinensis* (Sapindaceae) HSAUP194.21, living culture SAUCC194.21.

**Figure 7.**
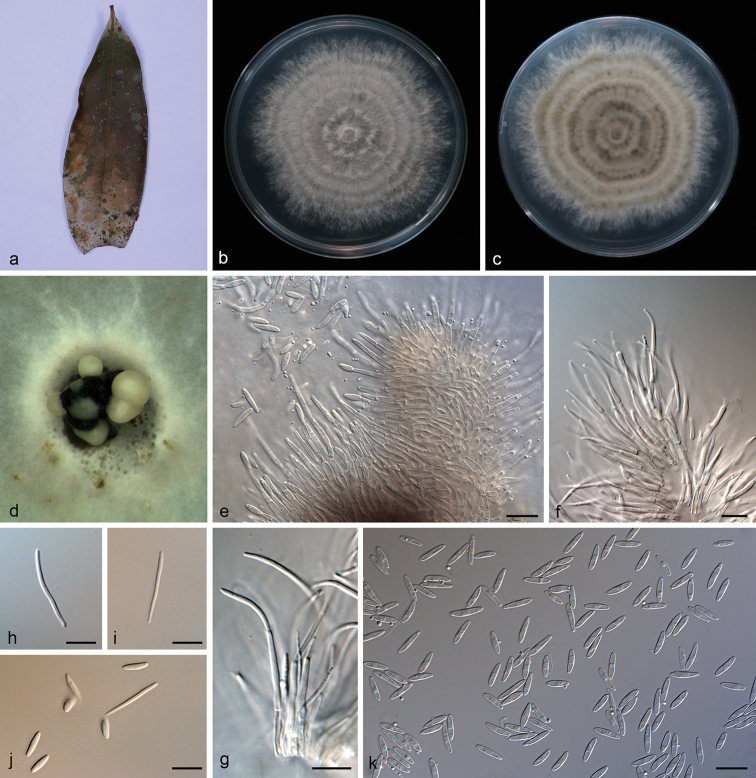
*Diaporthe osmanthi* (SAUCC194.21) **a** infected leaf of *Litchi chinensis***b, c** surface and reverse of colony after 15 days on PDA**d** conidiomata **e–g** conidiophores and conidiogenous cells **h, i** beta conidia **j, k** alpha conidia. Scale bars: 10 μm (**e–k**).

##### Notes.

*Diaporthe osmanthi* was originally described from the leaves of *Osmanthus fragrans* (Oleaceae) in Guangxi province, China (Long et al. 2019). In the present study, phylogenetic analyses (Fig. 1) indicated that the strain SAUCC194.21 is closely related to *Diaporthe osmanthi* and *D. podocarpi-macrophylli* (Gao et al. 2017). Morphological comparison indicated that this strain was most similar to *D. osmanthi* by the size of alpha conidia and beta conidia. We therefore identify this strain as belonging to *D. osmanthi*.

#### Diaporthe pandanicola

Taxon classificationFungiDiaporthalesDiaporthaceae

Tibpromma & K.D. Hyde, MycoKeys 33: 44 (2018)

6D78E7E2-75D9-50C1-A129-BC29A53C0A52

Figure 8

##### Description.

Asexual morph: Conidiomata pycnidial, 3–5 pycnidia grouped together, superficial to embedded on PDA, erumpent, thin-walled, dark brown to black, globose or subglobose, exuding white creamy conidial mass from ostioles. Conidiophores hyaline, aseptate, cylindrical, smooth, straight to sinuous, unbranched, aggregated, 17.0–26.5 × 2.0–3.0 µm. Conidiogenous cells phialidic, cylindrical, terminal, 10.0–20.0 × 1.5–1.8 µm. Alpha conidia hyaline, smooth, aseptate, ellipsoidal, eguttulate, apex subobtuse, base subtruncate, 6.5–9.0 × 1.8–2.5 µm (mean = 7.5 × 2.0 μm, n = 20). Beta conidia hyaline, aseptate, filiform, curved, tapering towards apex, base truncate, 26.0–32.8 × 1.0–1.6 µm (mean = 29.0 × 1.3 μm, n = 20). Gamma conidia infrequent, aseptate, smooth, straight, hyaline, 12.5–14.5 × 1.3–1.8 µm (mean = 13.5 × 1.6 μm, n = 6). Sexual morph not observed.

##### Culture characteristics.

Cultures incubated on PDA at 25 °C in darkness, growth rate 12.8–15.0 mm diam/day, flat, cottony in centre, with aerial mycelium sparse toward margin, white on surface, white to pale yellow on reverse.

**Figure 8.**
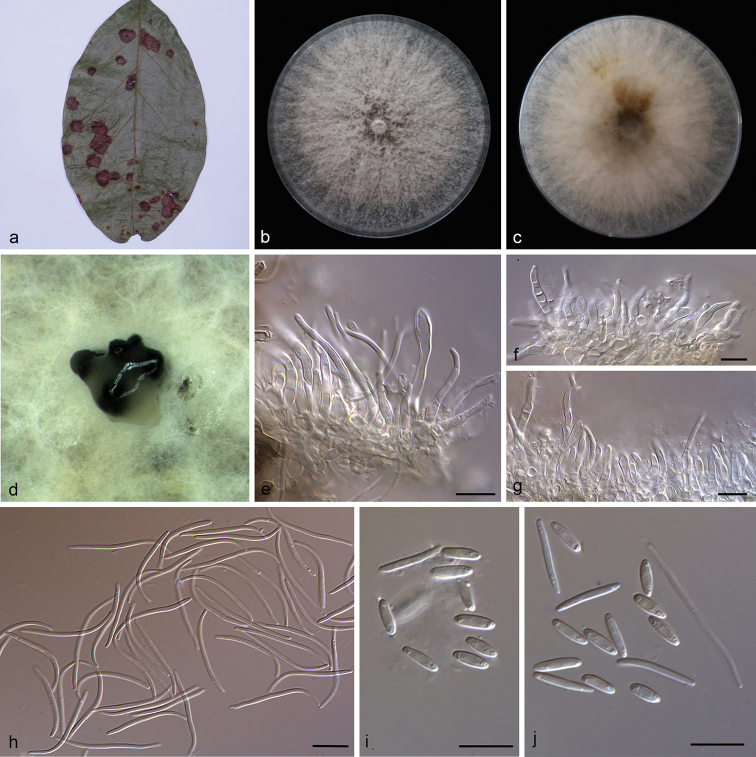
*Diaporthe pandanicola* (SAUCC194.82) **a** infected leaf of *Millettia reticulata***b, c** surface and reverse of colony after 15 days on PDA**d** conidiomata **e–g** conidiophores and conidiogenous cells **h** beta conidia **i** alpha conidia and gamma conidia **j** alpha conidia, beta conidia and gamma conidia. Scale bars: 10 μm (**e–j**).

##### Specimen examined.

China, Yunnan Province: Xishuangbanna Tropical Botanical Garden, Chinese Academy of Sciences, on diseased leaves of *Millettia reticulata* (Fabaceae). 19 April 2019, S.T. Huang, HSAUP194.82, living culture SAUCC194.82.

##### Notes.

*Diaporthe pandanicola* was originally described by Tibpromma et al. (2018) on healthy leaves of *Pandanus* sp. (Pandanaceae) as an endophytic fungus. Our strain (SAUCC194.82) is closely related to *Diaporthe pandanicola* based on phylogenetic analyses (Fig. 1). The differences of nucleotides in the concatenated alignment (19/533 in the ITS region and 11/351 in the TUB region) are less than 3%. Morphologically, our strain produces alpha conidia, beta conidia and gamma conidia, while *Diaporthe pandanicola* did not sporulate. We therefore identify our strains as *Diaporthe pandanicola*.

#### Diaporthe pometiae

Taxon classificationFungiDiaporthalesDiaporthaceae

S.T. Huang, J.W. Xia, W.X. Sun, & X.G. Zhang sp. nov.

D8C614FF-3589-5562-82FA-F44963136C2F

837815

Figure 9

##### Etymology.

Named after the host genus on which it was collected, *Pometia pinnata*.

##### Diagnosis.

*Diaporthe pometiae* is similar to *D. biconispora* but differs in having smaller alpha conidia (5.7–8.3 × 2.2–3.0 vs. 6.0–10.5 × 2–3.5 μm) and types of conidia (*D. pometiae* produces beta conidia unlike *D. biconispora*).

##### Type.

China, Yunnan Province: Xishuangbanna Tropical Botanical Garden, Chinese Academy of Sciences, on diseased leaves of *Pometia pinnata* (Sapindaceae). 19 April 2019, S.T. Huang, HSAUP194.72 holotype, ex-type living culture SAUCC194.72.

##### Description.

Asexual morph: Leaf spots subcircular, fawn to dark brown. Conidiomata pycnidial, subglobose to globose, aggregated in groups, black, coated with white hyphae, thick-walled, exuding creamy droplets from ostioles. Conidiophores hyaline, smooth, slightly septate, branched, densely aggregated, cylindric-clavate, straight to slightly sinuous, 22.5–32.5 × 1.0–2.0 μm. Conidiogenous cells 15.0–22.5 × 1.0–1.5 μm, phialidic, cylindrical, multi-guttulate, terminal, tapering towards apex. Alpha conidia abundant in culture, 2–4 guttulate, hyaline, smooth, aseptate, ellipsoidal to oblong ellipsoidal, with both ends obtuse, 5.7–8.3 × 2.2–3.0 µm (mean = 6.7 × 3.1 μm, n = 20). Beta conidia, hyaline, aseptate, filiform, multi-guttulate, slightly curved, tapering towards to apex, 27.8–34.5 × 1.0–1.7 µm (mean = 21.7 × 1.4 μm, n = 20). Gamma conidia not observed. Sexual morph not observed.

**Figure 9.**
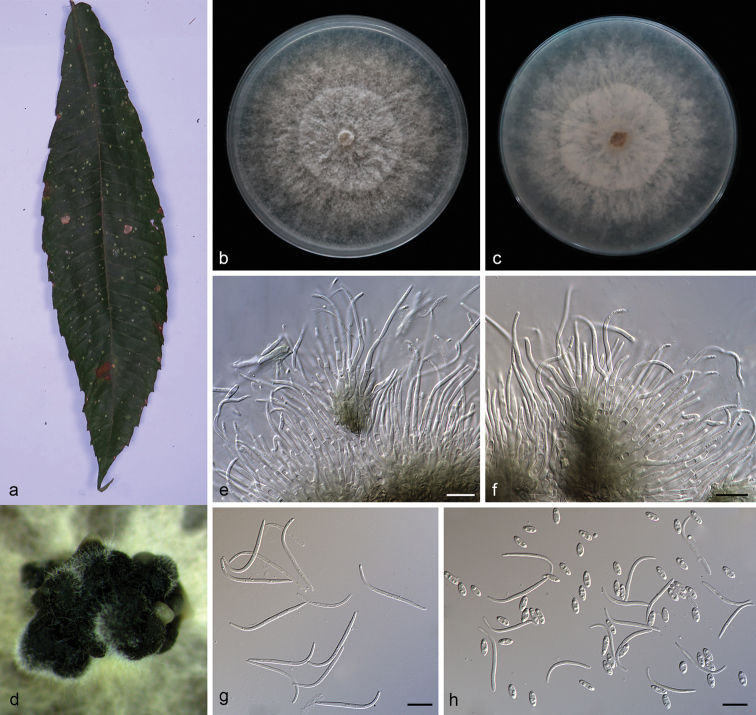
*Diaporthe pometiae* (SAUCC194.72) **a** infected leaf of *Pometia pinnata***b, c** surface and reverse of colony after 15 days on PDA**d** conidiomata **e, f** conidiophores and conidiogenous cells **g** beta conidia **h** alpha conidia and beta conidia. Scale bars: 10 μm (**e–h**).

##### Culture characteristics.

Cultures incubated on PDA at 25 °C in darkness, growth rate 11.5–13.0 mm diam/day, cottony with abundant aerial mycelium, with a concentric zonation, white on surface, white to grayish on reverse.

##### Additional specimens examined.

China, Yunnan Province: Xishuangbanna Tropical Botanical Garden, Chinese Academy of Sciences, 19 April 2019, S.T. Huang. On diseased leaves of *Persea americana* (Lauraceae), HSAUP194.19 paratype, ex-paratype culture SAUCC194.19; on diseased leaves of *Heliconia metallica* (Musaceae), HSAUP194.73 paratype, ex-paratype culture SAUCC194.73.

##### Notes.

*Diaporthe pometiae* is introduced based on the multi-locus phylogenetic analysis, with three isolates clustering separately in a well-supported clade (ML/BI = 100/1). *Diaporthe pometiae* is most closely related to *D. biconispora*, but distinguished based on ITS, TUB, TEF and HIS loci by 74 nucleotide differences in the concatenated alignment, in which 2/492 are distinct in the ITS region, 8/353 in the TUB region, 49/370 in the TEF region and 15/471 in the HIS region. Morphologically, *Diaporthe pometiae* differs from *D. biconispora* in its smaller alpha conidia (5.7–8.3 × 2.2–3.0 vs. 6.0–10.5 × 2–3.5 μm). Furthermore, *Diaporthe pometiae* produces beta conidia unlike *D. biconispora* (Huang et al. 2015).

## Discussion

The Yunnan Province in southeastern China has a unique geography where three climatic regions meet: the eastern Asia monsoon region, the Tibetan plateau region, and the tropical monsoon region of southern Asia and Indo-China. The environment is conducive to growth of unusual microbial species. Species diversity in Yunnan Province is high compared to other parts of China.

Previously, species identification of *Diaporthe* relied on the assumption of host-specificity, leading to the proliferation of names. The morphological characters of *Diaporthe* could be changeable, as most taxa in culture do not produce all spore states of the asexual (alpha, beta and gamma conidia) or the sexual morph (Gomes et al. 2013). Based on a polyphasic approach and morphology, more than one species of *Diaporthe* can colonize a single host, while one species can be associated with several hosts (Gomes et al. 2013; Gao et al. 2017; Guarnaccia and Crous 2017; Guarnaccia et al. 2018; Guo et al. 2020). These studies revealed a high diversity of *Diaporthe* species from different hosts. Our study supports this phenomenon. For example, *Diaporthe arecae* (SAUCC194.18) and *D. pometiae* (SAUCC194.19) were collected from *Persea americana*; In addition, isolates of *D. middletonii* were obtained from three hosts (*Litchi chinensis*, *Lithocarpus craibianus*, *L. glaber*). As for host specificity, in our study, four species of *Diaporthe*, *D. machili* (SAUCC194.69), *D. middletonii* (SAUCC194.27), *D. osmanthi* (SAUCC194.21), and *D. pometiae* (SAUCC194.72) were isolated from *Litchi chinensis* and *Pometia pinnata* belong to the Sapindaceae, and *D. litchiicola* also was reported from *Litchi chinensis* in Queensland (Tan et al. 2013); however, *D. machili* (SAUCC194.111) also was isolated from *Machilus pingii* (Lauraceae), *D. middletonii* (SAUCC194.45) from *Lithocarpus glaber* (Fagaceae), *D. osmanthi* (GUCC 9165) from leaves of *Osmanthus fragrans* (Oleaceae) (Long et al. 2019), and *D. pometiae* (SAUCC194.19 and SAUCC194.73) from *Persea americana* (Lauraceae) and *Heliconia metallica* (Musaceae). These results provide evidence that many species are able to colonise diverse hosts and several different species could co-occur on the same host. It seems obvious that specificity does not occur at the family level.

For the current study, sixteen strains isolated from ten host genera represented three new species and five known species, based on morphological characters and phylogenetic analyses of the five combined loci (ITS, TUB, TEF, CAL and HIS). The descriptions and molecular data for species of *Diaporthe* represent an important resource for plant pathologists, plant quarantine officials and taxonomists.

## Supplementary Material

XML Treatment for Diaporthe arecae

XML Treatment for Diaporthe chrysalidocarpi

XML Treatment for Diaporthe hongkongensis

XML Treatment for Diaporthe machili

XML Treatment for Diaporthe middletonii

XML Treatment for Diaporthe osmanthi

XML Treatment for Diaporthe pandanicola

XML Treatment for Diaporthe pometiae
